# Opportunities for Inclusion and Engagement in the Transition of Autistic Youth from Pediatric to Adult Healthcare: A Qualitative Study

**DOI:** 10.1007/s10803-022-05476-4

**Published:** 2022-03-09

**Authors:** Jennifer L. Ames, Arjun Mahajan, Meghan N. Davignon, Maria L. Massolo, Lisa A. Croen

**Affiliations:** 1 Division of Research, Kaiser Permanente Northern California, Oakland, CA USA; 2 Kaiser Permanente Roseville Medical Center, Pediatric Subspecialties; Regional Medical Director of Pediatric Developmental Disabilities, Roseville, CA USA

**Keywords:** Autism spectrum disorder, Healthcare transition, Autistic youth, Qualitative research, Patient-centered care, Pediatrics

## Abstract

Transitioning autistic youth from pediatric to adult healthcare requires coordination of multiple stakeholders, including youth, caregivers, and pediatric and adult care providers, whose interests at times overlap but often differ. To understand barriers and facilitators to inclusive transition experiences, we conducted thematic analysis of interviews with 39 stakeholders from the same large, integrated healthcare system. We identified three major themes: (1) Navigating the healthcare transition without guidance, (2) Health consequences of a passive healthcare transition, and (3) Strategies for inclusion and continuous engagement. Facilitators included gradual transition planning, a warm handoff between providers, and support of shared healthcare decision-making. Providers also sought clinical tools and logistical supports such as care coordinators and longer transition-specific visit types to enhance patient-centered care.

**Supplementary Information:**

The online version contains supplementary material available at 10.1007/s10803-022-05476-4.

A note on language used in this article: we use identity first language (i.e., autistic person), as opposed to person first language (i.e., person with autism) and sometimes refer to autistic youth as patients, given the context of this study in the healthcare system. We have chosen identity-first language in accordance with the preference of the autistic community with whom we work, though we acknowledge that some autistic people prefer person-first language.

Nearly 73,000 autistic youth transition to adulthood each year (Shattuck et al., 2018). Given that young people on the autism spectrum often have co-occurring medical and psychiatric conditions that require specialized and consistent health services, the healthcare transition from pediatric to adult care is a multifaceted and, consequently, vulnerable period for this population (AAP & AAFP, 2011; Davignon et al., 2018; Rast et al., 2020; Woodward et al., 2012). Research suggests that the healthcare transition for many autistic youth is substandard, often lacking individualized adjustments for specific sensory sensitivities and communication preferences, limited transition planning services, and inadequate provider training and awareness about autism (Ames et al., 2020; Cheak‐Zamora et al., 2015; Kuhlthau et al., 2015; Muskat et al., 2015; Nicolaidis et al., 2015). Further, autistic youth in the U.S. are less likely than peers with other mental, behavioral, and developmental conditions to have conversations about managing their own health during the transition period, with more than half of autistic youth and their families receiving few to no resources for building transition readiness (MCHB, 2019; Zablotsky et al., 2020). In comparison with autistic youth who do not receive healthcare transition services, those who do have increased satisfaction with their care and improved health outcomes, such as fewer emergency department visits, missed school days, and inpatient hospitalizations (Cheak-Zamora et al., 2013). Thus, transition planning benefits the long-term health and well-being of both autistic youth and their caregivers.

Several qualitative studies have explored barriers and facilitators of the healthcare transition of autistic youth and youth with other developmental disabilities (Cheak-Zamora & Teti, 2015; Cheak-Zamora et al., 2017; Franklin et al., 2019; Kuo et al., 2018). The key themes among studies on youth and caregivers have centered on young adult agency and health-related independence (Cheak-Zamora et al., 2017), tensions between the desires of autistic youth and their caregivers, and the myriad sources of concern and anxiety among families in navigating the transition process, including lack of supports across multiple service settings (Cheak-Zamora & Teti, 2015; Cheak-Zamora et al., 2017; Kuhlthau et al., 2016; Kuo et al., 2018). Studies among healthcare providers suggest that poor communication and cultural differences between pediatric and adult care teams create additional barriers to effective transitions (Franklin et al., 2019; Reiss et al., 2005). This literature underscores how transition interventions must address the needs and constraints of three major stakeholders—youth, their caregivers, and their pediatric and adult care providers—whose interests at times overlap but often differ.

Our study expands the healthcare transition literature by synthesizing the narratives of all three of these stakeholder groups within one large, integrated healthcare system. Affirming that the engagement of autistic youth themselves is critical to advancing inclusionary healthcare research (Post et al., 2013), this study seeks to place autistic perspectives and experiences at center stage in order to directly and accurately capture their healthcare transition experiences (Crane, 2013). Through thematic analysis of these transition narratives, we aim to identify opportunities and improvements to make healthcare more inclusive and accessible to neurodivergent individuals.

## Methods

### Recruitment and Eligibility

This study is set in Kaiser Permanente Northern California (KPNC), a large integrated healthcare system serving 4.5 million members, where youth seen in pediatrics are automatically assigned an adult primary care provider when they turn age 18. We recruited participants from three stakeholder groups using different sampling techniques, described below, in order to capture a wide range of experiences across provider type and patient age and gender.

Providers: We reached out to a subset of KPNC pediatric and adult primary care and mental health providers who indicated in a previous transition survey study (Ames et al., 2020) their willingness to be contacted for an interview. A large number of providers expressed interest on the prior survey (n = 225), and 10 were invited based on having transition-aged patients with an ASD diagnosis in their panel.

Autistic youth: We identified a pool of potential youth participants (N = 85) through (a) recommendations of twelve primary care physicians who had participated in the previous survey and worked in different KPNC medical centers and (b) families who participated in a previous autism study and had transition-aged (i.e., 14–25 years old) autistic children.

Caregivers: Caregivers of the invited autistic youth were also invited to participate in the interviews (N = 79).

Patients and caregivers received by mail an invitation packet with a cover sheet explaining details of the study and consent and assent forms. Providers were invited by phone or email. We conducted interviews with a total of 39 participants, including 7 autistic youth who participated alone (1 pre-transition, 6 post-transition), 7 pairs of autistic youth with their caregivers who were interviewed together (3 pre-transition, 4 post-transition), 10 caregivers who participated alone (3 had pre-transition children, 7 post-transition), and 8 providers (2 from adult medicine, 2 from family medicine, 2 from pediatrics, 2 from pediatric mental health).

### Procedures

Each participant took part in a semi-structured phone interview that ranged between 30 and 120 minutes. Following best practices established in other studies (Cheak-Zamora et al., 2017; Yin, 2017), our interviews began with pre-designed questions outlined in an interview guide (example included in Supplementary Table 1), and typically moved beyond them through probing and follow-up questions. Interview guide questions were co-created by three qualitative researchers, including a PhD-level anthropologist, (MLM), who also conducted the interviews, and members of Academic Autistic Spectrum Partnership in Research and Education (AASPIRE), with particular attention to making questions accessible to autistic participants by being concrete and specific without compromising the open-ended nature of the interview. Of the three trained interviewers, two interviewed autistic youth and caregivers via telephone. All the provider interviews were conducted by a third interviewer, a physician, (MND), who used their shared background to probe providers about their clinical experiences. The interview for youth was initially piloted with one autistic young adult who was a member of the KPNC Autism Research Program's Community Advisory Board (CAB) and modified based on his feedback. Each interview was audio-recorded upon obtaining verbal consent or assent from the respondent(s) and transcribed verbatim by a single professional transcriptionist. Non-physician participants were paid an honorarium for their participation. All study procedures were approved by the KPNC Institutional Review Board (IRB) and participants provided written informed consent prior to the interview.

### Communication and Facilitation Support for Youth

We used multiple strategies to ensure the interview process was accessible and that youth felt comfortable discussing their healthcare experiences with a stranger. We offered various participation modes (telephone, instant messenger, email, or in-person), and all participants chose to interview via telephone. We provided autistic participants a copy of the interview questions in advance, noting that they would also be asked follow-up questions. We encouraged autistic participants to take breaks during interviews. Finally, we provided the opportunity for youth to bring in a supporter if they desired, which some did. In other cases, a caregiver could be heard in the background providing answers, so they were invited and consented to be part of the interview on the spot.

### Data Analysis

In conducting our analysis, we viewed autism through the lens of a social model, recognizing that the autistic participants in our study were predominantly seeking primary care for reasons tangential to autism. In contrast to a medical model, which focuses on addressing personal impairments associated with the disability, a social model intends to redress social and environmental inequities for persons with disabilities (Haegele & Hodge, 2016).

We analyzed interview transcripts according to a thematic analysis framework to capture and interpret the experiences and perspectives in each stakeholder group (Guest et al., 2011). To ensure a rigorous and consistent analysis across groups, we further applied Braun and Clarke’s 6 phase approach to thematic analysis (Braun & Clarke, 2006). Three authors (MLM, JLA, AM) read every transcript three times to become familiar with the content, identify key themes and create an initial coding dictionary (Steps 1 and 2). We then coded the interviews, working independently to identify key features of the data (Step 3). Coding was an iterative process in which the team met four times to address discrepancies and build consensus in identifying additional themes (Step 4). Subsequently, we met once per week for 2 months to collate codes in search of key themes for each stakeholder group (Step 5). Thematic consensus was reached by the sixth session. The coding team created organizational matrices and visual thematic maps that were iteratively updated to ensure thorough data capture. We met twice to review and refine the themes in relation to coded extracts and the entire data set (Step 6).

## Results

**Fig. 1:**
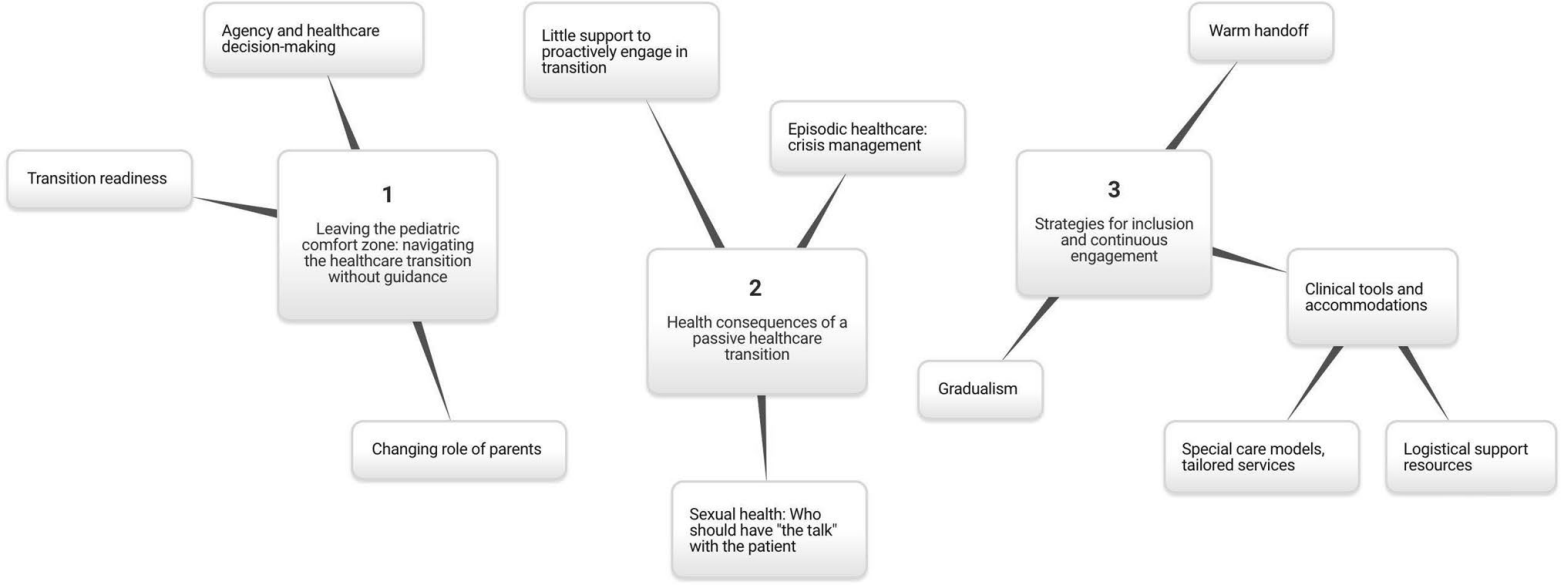
Thematic map of major themes and subthemes

Our analysis identified three major themes, each encompassing multiple subthemes as summarized in the thematic map (Fig. 1): Leaving the pediatric comfort zone: navigating the healthcare transition without guidance (Theme 1); Health consequences of a passive healthcare transition process (Theme 2); and Strategies for inclusion and continuous engagement (Theme 3). Though we focus on three themes in the space of this paper, the thematic map and Supplementary Table 2 reflect additional participant quotes and topics raised in the narratives.

### Theme 1: Leaving the Pediatric Comfort Zone: Navigating the Healthcare Transition without Guidance

For post-transition patients, the healthcare transition typically occurred automatically when they reached age 18 though many had not necessarily reached transition readiness. Youth and their caregivers often felt as if they had no roadmap to navigate the transition and the unfamiliar modes of adult care and healthcare decision-making. This theme highlights the unclear expectations for the transition among autistic patients, caregivers, and providers with respect to patient agency in healthcare decision making, assessments of transition readiness, and shifting roles of the caregiver.

#### Agency and Healthcare Decision-Making

During the transition, many youth anticipated progressive independence from their caregivers and control over the decisions that affect their bodies and well-being. Transitioning youth described feeling more assertive over their body autonomy, desiring greater control over who could examine and touch them in healthcare settings. While the healthcare system generally expects young adults to start making their own healthcare decisions upon turning 18, we found that, in practice, caregivers and providers struggled to extend this absolute independence to their autistic children and patients, respectively.

Youth narratives often focused on how disclosure of autism in adult care influenced patient-provider interactions, in some cases hindering and, in others, facilitating the patient’s exercise of agency. In the following quote, provider assumptions about autism resulted in an interaction that an autistic patient perceived as patronizing:

I didn’t really feel like he [adult provider] respected what I was saying. She [patient’s mother] told him about me being on the spectrum, and he took her aside and questioned the validity of my diagnosis…I felt like I was being talked down to a lot more than I was by the pediatrician…I wish I was you know, treated like an adult by an adult doctor. (Post-transition patient)

In a separate interview, disclosure of autism beneficially informed the patient-provider relationship by facilitating an exchange that ultimately helped transform a young adult’s relationship to their autism diagnosis as well as their healthcare:

I told her [adult doctor] how I feel about it [the autism diagnosis]; like how feeling different because of that. She brought my parents in and talked to them, so they can know… I felt comfortable enough to say how I feel…[Things] have gotten better, ‘cause someone’s finally understood. (Post-transition patient)

Others were also wary of how prevailing autism stereotypes might overshadow key parts of their identity and wished to affirm their identity as separate from their diagnosis:

“I don’t really stress it [autism diagnosis] too much. I really don’t think people that are diagnosed with something—that’s what shapes them. It’s just their diagnosis. You know? I would like them [new doctor] to know that …a lot of people that are on the autism spectrum are portrayed as nerds and I do play sports, so that’s not always true” (Post-transition patient)

Some caregivers viewed their child’s autism diagnosis as essential information for the adult care team, helping the latter to recognize and accommodate the patient’s communication style. Caregivers worried that not disclosing their children’s autism would result in adult providers not making necessary accommodations to allow their patients to self-advocate and receive the best care as adults.

You can tell that you have to be your own advocate [in adult care]. You don’t get the help that you need and they [the healthcare system] have to be aware that someone with autism… it’s pretty difficult [for an autistic individual] to self-advocate. (Caregiver of post-transition patient)

In these examples, both youth and caregivers emphasized that providers should know about the autism diagnosis—not for medical purposes or treatments—but for making the healthcare interaction more inclusionary for the autistic youth. This desire for accessible healthcare was echoed in other interviews with autistic youth, who appreciated when doctors spoke to them plainly and directly rather than turning to their caregiver during the healthcare appointment.

[in describing the ideal doctor] A doctor who knows, like, how to…understand, like, adults like with disabilities and…you know, basically, treat you the same. (Pre-transition patient)

While caregivers generally encouraged more direct patient-provider communication and their children’s independence in healthcare decisions, some were wary of relinquishing control and sought doctors who would facilitate some level of caregiver involvement:

I want somebody [an adult doctor] that’ll talk to [patient name] directly, not just to me. But I also don’t want someone that is going to let [patient name] make all the decisions. (Caregiver of post-transition patient)

Furthermore, many caregivers of youth with high support needs relied on conservatorship, in which the caregiver retains complete control.

We are trying to include him in all these decisions…They’ve asked him, “Are you okay with your mom, being here, making these decisions?”…I’ve talked to him about the conservatorship, and I’ve said, “This is so we can protect you from making decisions that are bad for you.” So, he’s okay with all that. He understands that we’re looking for his best interests, and we we’re not trying to take away any independence that he might have. (Caregiver of post-transition patient)

In another interview, a provider recounted their uncertainty in a situation involving a conservatorship and sterilization. The provider, concerned about the healthcare decision, spoke directly with the patient in an effort to confirm that the young adult fully understood the ramifications of sterilization. Other providers opted for a more cursory role in these decision-making arrangements and did not engage beyond raising awareness that these were legal topics to be explored by the family.

I think with any [patient] turning 18 there’s that privacy…I’ll just bring it up that thing; you know, that, “There’s legal change that happens after 18.” And fortunately, I think the families that have gone through that process have been savvy enough to kind of navigate that on their own. (Adult provider)

More flexible and empowering healthcare decision-making arrangements that support greater patient autonomy such as supported decision-making did not commonly come up in interviews. However, constraints of the healthcare system appeared to pose pragmatic challenges for families wishing to establish these alternatives to conservatorship. For example, one family wanted a shared healthcare arrangement but could not find a straightforward way to make electronic communications over the patient portal go to both the patient and caregiver.

#### Transition Readiness

Stakeholders had different interpretations of what constituted transition readiness. For many youth, transition readiness was a stage inevitably reached by turning 18 rather than by acquiring a concrete skillset. Most youth spoke of transition skills in vague terms but desired to practice these skills so that they could prepare for the expectations of adulthood. One pre-transition patient appreciated how an earnest conversation with his pediatrician about entering adulthood with autism was important to his transition preparation:

He [pediatric provider] was talking about, “Do you know what your disability is?” How do you feel that you have this disability? And how do you think life is going to be for you with this? […] It was interesting. I mean, it wasn’t one of the best conversations but later on, it was probably time to talk about this, too. (Pre-transition patient)

Despite optimism and curiosity about being treated as an adult and having more independence, many youth did not know what to expect in adult care.

It was kind of strange. I didn’t really know that much about that I was changing to a different doctor, but it’s a lot more independent. I don’t know. I get to do it myself—sign up and talk to the doctor myself—instead of having a parent hovering over. (Post-transition patient)

This lack of guidance could later manifest as ambivalence after the transition, as expressed by one youth, “I would see my pediatrician, if I had the chance.” (Post-transition patient).

Caregivers were apprehensive about the timing of the transition at age 18 and questioned whether their child had sufficient interest in and preparation for taking charge of their personal health. Several caregivers wondered if the healthcare system could accommodate factors like developmental delays in its determination of transition readiness.

Because really, even though they have a certain age … the assumption is based on that, when you become 18, you arrive at this set of knowledge that makes you adult. These kids don’t have that. (Caregiver of pre-transition patient)

Providers, meanwhile, did not have standardized tools or approaches for assessing transition readiness, relying instead on discussions with the patient and family. However, these discussions typically only happened if the caregiver brought up the topic during the healthcare visit and had the most value if sustained over several appointments.

I don’t think you could probably assess that [transition readiness] with one visit. I think you have to kind of get to know the patient. And you probably have to rely a lot on the family that’s with them. (Adult provider)

#### Changing Role of Parents

Some parents recognized the move from pediatrics to adult care as a time for their own transition from healthcare manager to healthcare supporter. However, the mechanics of this shift were stressful for parents, who wanted to step away but also felt they needed to stay involved in order to mediate communication and assist both their child and the provider in managing their child’s healthcare.

I’m sure they’re going to give him the option to have me wait outside … it’s just perfectly fine… Because he is going to be an adult; he needs to learn how to do things on his own…But I just want to make sure that he would be able to understand all the questions (Caregiver of pre-transition patient)

This tension was understandable for caregivers, who had often been in the position of being more expert about autism than their child’s providers and felt little active support in planning for the healthcare transition. Continuing to help their children in early adulthood also made some caregivers worried about their children’s future and whether they would develop the capacity for self-advocacy.

I’ve ended up being a crutch for her [daughter] because it’s too much to shove at her at once…If [medical facility] could figure out a way to make it a more incremental change because, you know, someday I’m not going to be here … I would like her to learn some of these skills so that she can advocate for herself a little bit, you know. (Caregiver of pre-transition patient)

Many patients expressed feeling more comfortable when their parents joined the medical appointments, especially in the beginning of their transition to adult care. However, accommodations for these rooming preferences were not always straightforward and, without preparation, could precipitate confusion and unpleasant healthcare experiences for all stakeholders, particularly in the initial appointment.

[T]here would be situations where a staff wouldn’t recognize that, “Oh, this is a special needs patient. Family needs to be in the room.” And they would ask the parent to wait outside. And then, you have this patient that’s not necessarily the most comfortable just meeting with a physician one-on-one. And the parent would become very distressed, and there would just be a very—unhappy first encounter. (Adult provider)

Caregivers also described a tension between being positive about their child’s growing independence and concern over relinquishing control, especially in light of unique medical needs and safety concerns, including medication management and co-occurring conditions such as epilepsy and ADHD.

### Theme 2: Health Consequences of a Passive Healthcare Transition

Stakeholders described a passive healthcare transition process with little support to proactively engage in the transition process. This passivity manifested in two problematic healthcare patterns, (a) an episodic model of adult care defined by crisis management rather than regular preventive care, and (b) neglect of sexual health topics, both of which may make autistic youth more vulnerable to certain adverse health consequences in adulthood.

#### Little Support to Proactively Engage in Transition

In the experiences of several youth, the transition process “just sort of happened” (Post-transition patient) without notable conversations or preparation, resulting in a sense of disengagement:

It [healthcare transition] was just sort of an abrupt change… there was a conversation, but I didn’t really follow it completely and I didn’t really have that much of a care about it. (Post-transition patient)

Several caregivers were caught off-guard by the discontinuity of behavioral and educational services and supports during the transition to adulthood and were anxious about the implications for their children, especially with regards to the loss of structure and social engagement. Caregivers also felt inadequately prepared to manage the transition process and sought more active engagement from the healthcare system, beyond “paper handouts coming home. I don’t call that healthcare” (Caregiver of pre-transition patient). They worried that autism would make it challenging for their children to suddenly shift to scheduling their own appointments, effectively and accurately communicating health issues with their adult doctors, and remembering next steps after the appointment. Some caregivers relied on the healthcare system for cues regarding what actions to take, with regards to conservatorship or other arrangements, in order to assist their transition-age children in healthcare matters.

[W]e understand that we need to sign some forms so that we can continue to help facilitate his healthcare, and nobody’s told us what that looks like. (Caregiver of pre-transition patient)

Adult providers, too, were not always sure how to engage in the transition process, especially without more supports.

It probably will be helpful to have some kind of support system to help the pediatricians. There isn’t much that we can do on the adult medicine side, other than just wait for these patients to somehow show up on their doorstep. (Adult Provider)

Pediatric providers named several challenges they faced in navigating the transition process (Supplementary Table 2). These included difficulty finding opportunities to discuss the transition with patients ahead of time, especially if the patient did not visit their general pediatrician regularly or tended to go to a specialist for their routine care. Providers also did not always know when a patient transitioned because it would happen automatically in the system. It could also be difficult to find time to discuss transition planning during standard 20-min visits, where the focus tended to be on the most pressing medical needs. While providers were interested in resources that would help them identify and foster earlier, pro-active engagement with transition-age patients, they were also wary of the diminishing returns of too many alert systems demanding their attention.

#### Episodic Adult Healthcare

Unlike pediatric care, in which routine annual visits were the norm, narratives described adult care as episodic and reactive, with patients typically seeking care only when ill. Autistic patients and their families also perceived the dynamic in adult care as less about relationship-building and more about crisis management, which could be discouraging and lead to missed opportunities for preventive care in adulthood.

One of the things about adult medicine is that there’s not the routine visits…for somebody with developmental disabilities…To say, you know, “We’re going to see them twice a year no matter what.”…It also gives an opportunity to come in when there’s not a crisis…it’s like only going to the dentist if you have a tooth infection or root canal. So, it’s all that negative connotation… I think it makes it easier and better for the doctor and the patient to have that kind of a relationship. (Caregiver of post-transition patient)

Negative experiences in adult care could make it harder for autistic patients to stay engaged with the healthcare system and their primary care provider, in turn, potentially leading to delays in routine screenings, missed windows for early intervention, and poorer management of co-occurring chronic health conditions. Opportunities to build rapport with a new doctor over several visits would further help some young adults feel more comfortable on their own.

If I’ve had that doctor a few times. They know me. I know them a little bit. So, I know what’s coming and I feel like I can be a little bit more expressive. It’s normal, when you know people more, you tend to show your emotions more often. (Post-transition patient)

#### Sexual Health: Who Should have “The Talk” with the Patient?

In the passive transition process, discussion of key adult health topics, such as sexual health, were often neglected. Patients, caregivers, and providers alike expressed discomfort or uncertainty in broaching discussions about sexual health, further increasing the likelihood that these topics would fall through the cracks during the transition.

Parents and providers struggled to identify the point person to initiate the conversation. One parent highlighted that autistic teens may not be getting this information through usual channels like school and looked to providers to help fill the gap.

No one’s really had “The Talk” with him. We tried to pass it off on the school…I do wish that there was some sort of way the doctors could kind of broach sexuality…with these kids because of the fact that it’s already a difficult subject. And when you throw autism into it, it can get really tricky. (Caregiver of post-transition patient)

Another parent described feeling like a “nervous wreck” leading up to the conversation and was relieved when her daughter’s gynecologist took extra time to discuss sex and pregnancy (“It was a very positive experience”). Providers also noted the topic could be stressful for families and difficult to broach without a roadmap.

I just encouraged, you know, maybe she [the patient] talk with, you know, her OB/GYN… that creates a lot of anxiety, sometimes, for some of these patients and their parents. Like, “Oh my goodness! How do I explain this to my child?”… there’s no guidelines, I don’t think, about how you approach a family about that. (Adult provider)

As a consequence, autistic youth may be unprepared in adulthood to recognize and engage in healthy sexual relationships and navigate experiences of sexual victimization and abuse.

### Theme 3: Strategies for Inclusion and Continuous Engagement

Considering the challenges raised in these narratives, interviewees endorsed strategies that would support inclusion and more continuous engagement of stakeholders in the transition process. These strategies included more gradual timing of the transition, supports for the warm handoff between pediatric and adult care, and other recommendations for transition-focused clinical tools and accommodations.

#### Gradualism

In contrast to the abrupt transitions experienced by some autistic youth, those with positive experiences described transitions with manageable pacing and stability.

I’ve certainly been in there every year of my life, but it’s basically gone on—the transitions have all been slow. There hasn’t really been a significant step. It’s just been very steady. (Post-transition patient)

Caregivers suggested that starting transition planning a few years earlier would facilitate their child’s engagement in the process, including choosing and getting to know their new doctor, and sustain a person-centered healthcare experience.

I hope there is a bridge between, you know, when the transitioning is happening … between the pediatrics care and adult care… I want them to start, like, a little slowly and then, you know, convert completely into the adult care…I want them to know…the history of [name of patient] and everything. Yeah. I feel, you know, they have to be, like, little friendly and they know him for what he is and everything. (Caregiver of pre-transition patient)

Providers also noted the benefits of starting transition conversations with families earlier and one suggested that there could be some flexibility in delaying the age of transition in special circumstances.

#### Warm Handoff

The handoff between pediatric and adult care was identified as a critical opportunity for proactive communication, not just between doctors but also between the doctors and the family. Caregivers who received guidance and initiative in transition planning from their child’s pediatric provider spoke very positively. These benefits were particularly felt when the pediatric provider could help recommend a new doctor and broach transition topics with the patient over the course of several visits.

A warm hand-off from a provider who’s taken care of that patient with specific information about—experiences or triggers or just details that make a person a person—are (sic) more important to give a sense to the social interaction issues. (Adult Provider)

I don’t want a token support. I want real support that there’s some kind of meaningful sharing of information…why can’t this patient be at the center of the practice group that brings two silos together and bridges a silo between pediatric and adult care?…because you really need that liaison between those two worlds. (Caregiver of pre-transition patient)

The handoff was described in different ways (Supplementary Table 2). Providers envisioned systems such as a “roundtable” session with the patient, pediatrician, and new doctor or a “transitional call” to families in which they could introduce the adult doctor and address key aspects of the transition. As noted by parents, reassurance and support from the pediatric provider could help instill confidence in the youth as they embarked for adult care. Some parents expressed that personal touches and kindness by pediatricians could leave a big impression on families.

[The Goodbye letter] was congratulating him for kind of graduating from the child pediatric—the child psychiatry to adult psychiatry, and how much she enjoyed working with him, and good luck with your next one…it was all like that, really positive.” (Caregiver of post-transition patient)

#### Clinical Tools and Accommodations

##### Logistical Support Resources

Participants suggested several resources and improvements for the transition that would mitigate some historical systemic barriers (Supplementary Table 2) and “take the anxiety out [by letting autistic patients] know what to expect”. Many of the ideas highlighted generic tools and practices that could be built into the system and benefit all youth, including autistic youth. These logistical resources included appointment preparation checklists for youth to complete ahead of time, short instructional videos about the transition, and a portable medical portfolio that the patient could bring with them. Parents were also interested in how medical chart notes could inform new providers and staff of their child’s communication style.

Providers felt constrained by the rigidity in the booking system with its pre-specified appointment lengths and desired options for special appointment types like transition visits or longer, get-to-know-you visits. Such flexibility would relieve the pressure in their tight schedules so they could spend adequate time with new patients without the stress of taking time away from their next patient.

##### Tailoring the Resources/Transition Readiness Materials to Autism, When Needed

Youth conveyed very positive experiences when they transitioned to doctors who took a whole-person and long-term approach to their healthcare management.

“If I go with a problem, he’s [adult provider] not just addressing my immediate concerns. He’s nice. He kind of shows a commitment to long-term help and helping me with a healthier… life in general.” (Post-transition patient]

While providers desired to help their autistic patients thrive, both in health and in their communities, they did not always feel prepared to help their autistic patients navigate some of their most pressing transition needs, including disability support in higher education or employment programs. Caregivers of post-transition youth listed resources that they thought their children would have found helpful during the transition, including a class or video series that could build readiness (Supplementary Table 2). Caregivers and providers, alike, emphasized that a list of providers with autism experience would be very helpful and suggested that a transition care coordinator would be value added. A coordinator would be better equipped to pro-actively engage in the youth’s healthcare transition and could help navigate the transition across general and specialty care, a scenario encountered by several families interviewed. Overall, caregivers described an optimal transition approach as being person-centered and having a flexibility of options.

You have to, with the spectrum as it is, have all kinds of [transition] options prepared and to say, “Okay. Which is going to be the better, most effective, more meaningful to this particular individual? (Caregiver of post-transition patient)

## Discussion

Each group of stakeholders—autistic youth, their caregivers, and their healthcare providers—described distinct constraints during the transition process. Autistic youth were in various stages of readiness to manage their own care when they turned 18, with many feeling uncertain about what to expect from adult care. Caregivers, uneasy about their evolving roles, struggled to trust the system and worried that their maturing children would not be understood or adequately cared for in adult care. Pediatric and adult providers both described lack of time and specific training as barriers to transition planning and productive communication between the forwarding and receiving care teams. Taken together, these perspectives demonstrate a passive transition system without clear points of engagement for these three stakeholder groups. The narratives highlighted opportunities for improvements to the healthcare transition process that would encourage self-advocacy, support greater independence of transition-age and adult autistic patients, and foster long-standing patient-provider relationships in primary care.

Participant narratives, implicitly or explicitly, espoused person-centered, flexible approaches to support inclusion and continuous engagement in transition, including gradual transition timing, transition-oriented visits, and proactive communication among providers. In these healthcare narratives, it is important to appreciate that patients were typically seeking care not for autism but instead for other co-occurring conditions or routine preventive care. Consistent with a social model of neurodiversity (Haegele & Hodge, 2016) as opposed to a medicalized model of deficits, youth and caregivers desired providers with knowledge of autism, not to treat autism, but in order to find care teams that could recognize and accommodate the patient’s identity, expressive characteristics, and communication style, and more effectively care for the issue(s) that brought the patient into the office in the first place. Thus, the broader population of neurodivergent youth aging into adult care would also benefit from these opportunities to make the healthcare transition more inclusive and tailored to individual communication and developmental needs.

Autistic youth were interested in having more independence in the healthcare visit but did not always have adequate preparation and support in building transition readiness. Youth were interested in having broader conversations about adulthood with their provider and wanted opportunities to control their own autism narrative without their providers turning to autism stereotypes to assume their abilities and needs. These findings are in line with other qualitative studies from the patients’ perspective (Cheak-Zamora & Teti, 2015; Nicolaidis et al., 2015), which highlighted the resilience of autistic youth following experiences of feeling marginalized in healthcare visits (Cheak-Zamora et al., 2017). Further, while youth desire to assert their independence, they often have little understanding of the transition, which may contribute to low self-efficacy during the process (Cheak-Zamora & Teti, 2015).

Caregivers, meanwhile, typically saw few options beyond conservatorship as the path toward ensuring the best care for their child, indicating they received little support in learning how to recognize and facilitate their child’s self-advocacy in the healthcare system. As demonstrated in our study and others, caregiver confusion, concern, and stress about the process can make it challenging to shift into a social model of disability that would better support their child’s independence (Cheak-Zamora et al., 2017; Cheak‐Zamora et al., 2015). Compounding the problem, providers are also not always experienced with having both the patient and their guardian in the room and feel like they are treating two patients (Warfield et al., 2015). Empowering relationships among patients, the healthcare system, and caregivers, could help promote the youth’s independence while accommodating some degree of parental help into adulthood (Cheak-Zamora & Teti, 2015; Cheak-Zamora et al., 2017). For example, self-advocate groups have outlined frameworks for supported decision-making, in which patients designate a supporter to help them be informed about health-related matters, with the goal of maximizing autonomy while improving health outcomes (Network, 2014).

The problems of a passive healthcare transition suggest the need for further exploration of how to address system-level barriers and introduce active and engaging points in the transition process for all stakeholders. The lack of comprehensive or integrated services, including transition planning resources for autistic individuals, is a recurrent theme in the literature (Kuhlthau et al., 2016; Sosnowy et al., 2018). Healthcare providers, however, feel hindered to deliver individualized transition planning and support, in part because of logistical constraints (Anderson et al., 2018) such as incentive structures that do not accommodate the additional time these conversations take (Warfield et al., 2014). Likewise, caregivers need, but often do not receive, preparation and assistance during the transition (Cheak-Zamora & Teti, 2015). Thus, the transition is commonly perceived by families as abrupt without availability of adult support services (Warfield et al., 2015), not just in healthcare but also in other areas including employment and post-secondary education (Sosnowy et al., 2018). Many autistic young adults encounter poor person-environment fits in many educational and vocational services during the transition (Anderson et al., 2018; Briel & Getzel, 2014; Giarelli et al., 2013). In our study, a similar mismatch was apparent between the episodic, reactive model of adult care and the developmental needs of young autistic adults. Autistic youth desired some aspects of the adult model of care, such as being talked to directly, but would be better served by patient-centered improvements to care delivery, including more regular interactions with their provider.

Our study further highlighted how each stakeholder group, through discomfort or uncertainty, contributed to either avoidance or omission of sexual education during the transition. Such conversations are important opportunities to reinforce the sexual identity-affirming aspects of entering adulthood but are typically lacking for autistic youth in healthcare settings (Bennett et al., 2018; Cheak-Zamora et al., 2019; Holmes et al., 2020). This lack of sexual knowledge may place autistic adults at higher risk than non-autistic adults of sexual abuse and engagement in sexual experiences that are unwanted or later regretted (Pecora et al., 2016, 2019; Sevlever, 2013). Furthermore, some patients may be making decisions about sterilization and other aspects of their reproductive care without adequate sexual education (Roden et al., 2020).

Our analysis highlights key inflection points in the transition that contribute to higher healthcare satisfaction and are worthy of more attention, such as early initiation of transition planning in pediatric care and a warm handoff between pediatric and adult providers. Stakeholders noted patient-centered qualities and lasting impressions of these transition-focused conversations, and how they contributed to the patient gaining greater health literacy and developing a deeper connection with their healthcare provider. Previous literature supports the notion that person-centered planning in adult services and programs enhances the transition process for both the individual and their providers (Anderson et al., 2018), and that building transition readiness earlier in adolescence can mitigate the experience of abruptness (Kuo et al., 2018). The warm handoff may be a particularly important means of establishing good communication between the pediatric and adult care provider, building trust with a new provider (Cheak-Zamora & Teti, 2015), and priming the adult primary care relationship for success (Warfield et al., 2015; Zerbo et al., 2015). Whether or not the pediatricians are prepared for this role, families often look to them for guidance and direction in the transition. Thus, pediatricians can be particularly influential in the transition by helping families set expectations (Kuo et al., 2018), through both guiding with an encouraging tone and creating a sense of closure, as was noted by several families in our interviews.

Stakeholders emphasized the need for both generic and individualized supports to facilitate a proactive transition process. Many of the recommendations, including medical summaries, transition-specific appointments, transition checklists, and transition training for individuals and families, are recognized best practices for the transition of youth more broadly (Kuhlthau et al., 2015) and are being implemented in other healthcare settings (Harris et al., 2021). Logistical accommodations in the scheduling and reimbursing systems would also alleviate barriers that have historically disincentivized providers, especially mental health providers, from working with autistic adults (Warfield et al., 2015). Reinforcing the need for autism-focused transition supports for families (Anderson et al., 2018; Cheak-Zamora & Teti, 2015; Warfield et al., 2015) and primary care providers (Sohl et al., 2017), our stakeholders emphasized the value of specialized training to increase provider knowledge and sensitivity in caring for autistic adults, maintaining lists of adult providers with an interest in autism that could be shared with families, and implementing transition care coordinators. These accommodations would provide further scaffolding for the transition and facilitate the person-centered, flexible approaches that are needed if autistic patients are to receive the optimal healthcare they deserve.

Strengths of this study include the integration of three groups of stakeholder perspectives with representation across different points in the transition which helped to provide a balanced perspective of the transition experience. Further, recruiting all participants from a single, large healthcare delivery system allowed us to better distinguish system-level from individual-level constraints in the transition process. The thematic analysis was also conducted by three researchers, two of whom were not involved in the interviews, and reinforced an objective reading of the transcripts.

Our study has some limitations. While inclusive of multiple stakeholders within a single integrated healthcare system, our study is not necessarily representative of the experiences in different healthcare settings in the U.S. or across the autism spectrum. For example, our sample does not include non-speaking autistic youth, though we included some of their parents. We also did not explore how socioeconomic, racial, and cultural factors can shape healthcare experiences and more work is needed to address known disparities in access to transition resources (Eilenberg et al., 2019).

## Conclusion

Three different groups of stakeholders, each with different needs and experiences in the transition process for autistic youth, described a passive transition process with missed opportunities for engagement, communication, and preparation. Stakeholders suggested that changes such as shared healthcare decision-making models, earlier start of transition planning, and transition-focused healthcare appointments would support inclusion of autistic youth and help them and their families successfully navigate the transition. Providers also sought clearer protocols, greater time, and additional training to facilitate guiding their autistic patients and caregivers through the process. To address the need for person-centered care models, KPNC and other large healthcare systems are developing transition protocols, integrated within the electronic health record that can practicably be implemented in large, dispersed healthcare settings.

## Supplementary Information

Below is the link to the electronic supplementary material.

Supplementary file1 (DOCX 43 kb)
