# Supplementary material for: Opportunities for Inclusion and Engagement in the Transition of Autistic Youth from Pediatric to Adult Healthcare: A Qualitative Study
Source: J Autism Dev Disord. 2022 Mar 9;53(5):1850–61. doi: 10.1007/s10803-022-05476-4 (PMC10123038; doi:10.1007/s10803-022-05476-4)
Supplement: Supplementary file 1 — Supplementary file1 (DOCX 43 kb) [file 10803_2022_5476_MOESM1_ESM.docx]

**SUPPLEMENTARY MATERIAL**

Ames, et al. Opportunities for inclusion and engagement in the transition of autistic youth from pediatric to adult healthcare: A qualitative study

**Supplementary Table 1: Interview guide examples^1^**

| **Post Transition - Patient interview Guide:** |
| --- |
| **Introduction**  (This interview will be conducted using IM/Chat) First I will give you the purpose of the study and then I will ask you the interview questions. This interview will last approximately 45 minutes and I will ask you XX main questions with the possibility of a few follow-up questions. If you want to take a break at any point of the interview please let me know and we can stop.  **Purpose:** The purpose of this study is to learn about things that make it harder or easier to change from pediatric to adult care for young people with ASD. Things we will talk about in the interviews are, but not be limited to: quality of care, attitudes/beliefs, language/literacy, involvement in care, healthcare decision making process, education/income, stigma, family functioning. We will be interviewing patients, parents/caregivers.    **Interview Questions**  Introduction:  I am going to ask you questions about some of your experiences going to the doctor and conversations you may or may not have had with your doctor. I hope during this interview you will share your stories with me rather than one-word answers. There are no right or wrong answers, I just want stories and opinions. If there is anything you don't want to answer, this is OK.  I understand you have autism (spectrum disorder). What do you wish your doctor knew about how being on the spectrum affects your healthcare? Other areas of your life (what you eat, don’t eat, how you communicate with people etc)?  Now I want to talk about your pediatrician, what was his/her name? What was the name of your pediatrician? Your current doctor?   1. Please tell me what it was like for you when you changed from care by your pediatrician to care by your current doctor? (from pediatric care into adult care)    1. Tell me about any information given to you about how to make this change in doctors??    2. Tell me what kind of planning did you do with your doctor to help with this change? With your family?    3. Tell me about any planning you did with your pediatrician about the best time (age/ time of year) to change to your new doctor(s)?    4. How did you decide who your new doctor(s) should be?    5. What kinds of tools/ resources did your doctor give you to help with this change in care?       1. Examples: information about insurance, taking care of yourself, taking care of your healthcare needs, places to find support in the community       2. How do you like to get information (on-line, handouts, , etc)? 2. Do you remember your first visit with the doctor you have now (Dr. Name)? If so, think about your first visit (Dr. Name)?    1. Can you give me some examples of things you did to plan or prepare for you visit (e.g. talk to your pediatrician, work with your parents to make a plan)?    2. Is there anything that you wish your doctor would do differently that would make going to visit him or her better?  - How did the clinic contact you before your first visit? - Do you think your new doctor knew you had an Autism Spectrum Disorder? - Were you asked how much you know about Autism Spectrum Disorder? - What type of information did the clinic/doctor give you before your first appointment? After? (information about the clinic, information about insurance resources or ways to manage your health ) - How did your doctor discuss your medical history with you? - Tell me about any talks you had with your doctor about your healthcare needs? Goals? Planning to meet those goals? - Tell me about any talks you had with your doctor about the way you like to communicate (i.e. talking, texting, email, etc). - Tell me about any talks you had with your doctor about your ability to read and understand written information  1. Tell me about any other resources you have used to help you change doctors? E.g. websites, community groups, seminars, etc.    1. What was most helpful? 2. How important was it for you to prepare for your change to an adult doctor? (visual scale 0(not) to 10 (very). Tell me more about that.    1. Tell me about what went well when you changed doctors:    2. What did not go very well?    3. Is there anything you would have changed? 3. How well prepared were you to transition to adult care? (visual scale could be used) 4. If you were 100% in charge of how your transition went and could have anything that you wanted, what steps would you make happen? |

| **Pediatric Provider Interview Guide:** |
| --- |
| **Purpose:** The goal is to determine barriers and facilitators of a smooth transition from pediatric to adult care for ASD patients. We will be interviewing KPNC providers (adult primary care, pediatric primary and sub-specialty care, mental health) parents, and teens/young adults. The interview will take 45-60 minutes. This study was approved by the Kaiser IRB.  **Interview Questions**   1. Please tell me a little about your training and current medical practice 2. Describe any policy your clinic has for transitioning teens and young adults with special health care needs, including ASD, to adult healthcare services    - Tell me about any general policy/statement you have which describes the process?    - If there is a policy, how are you encouraged to share that information with your patients?    - how are staff members educated on the practice’s approach to transition?    - How does your clinic identify adult providers who are interested in caring for young adults with ASD? 3. Describe the process you use yourself to identify patients with ASD (or special health care needs) who will be transitioning into adult care?    - Registry? Flow sheet for tracking progress? 4. Describe a recent experience you have had with working with a child with ASD (or SCHN) who is in the process of transitioning/ recently transitioned: 5. How do you asses how well-prepared your patient is to transition?    - How do you assess his/her understanding of his/her condition (strengths, challenges)    - Needs and goals in self-care?      1. Make a plan to address needs, reach goals?    - Ability to be a self-advocate and access information?    - If needed - How did you help families understand changes in legal issues around decision-making (.e.. conservatorship/ supported decision making). Privacy?    - Knowledge about supports available (provider/ parent/ child)    - How does the presence of ASD affect this process? Intellectual or communication challenges? 6. Describe the steps you have taken/planning to take to help this teen/young adult and his/her family plan for a transition into adult care?    - How do/did you determine with them the optimal timing of transition?    - How do/did you help your families identify an adult primary care? Specialty care providers?    - How do/did you help the family go through process necessary for legal changes? (SSI, DD, conservatorship, etc)    - Tell me about any tools or resources you are using/used to assist in managing the transition process?    - Tell me about any tools/ resources you are providing/ provided family to help with the transition process? (e.g. Information about insurance, self-care management, community supports, etc)      1. What type of media do you use (on-line, handouts, etc)    - What is going/went smoothly?    - What kinds of barriers exist/existed?      1. Lack of interested/ knowledgeable adult providers      2. Time      3. Human resources (knowledgeable staff who can help with process)      4. Family not wanting to transition/ breaking bond between pediatric provider/ family    - Typical? If not, why not? 7. Describe any differences in the transition process for youth/ young adults with ASD compared to youth/ young adults with SHCN [optional] 8. **How confident do you feel in helping your families navigate the transition process?    - Facilitators? Barriers? 9. How do you address concerns a youth patient may have when transferring to a new adult provider.    - Differences in the way care is delivered between pediatric and adult care (e.g. specialists often consultants, don’t manage long term)? 10. What are some of the final preparations you make to transition a child to adult care?     - Making appointment     - Preparing summaries and emergency plan     - Communicating history/ plan of care to adult provider(s)     - Other records 11. How do you confirm whether transition has successfully take place? E.g. checking in with adult provider(s) to see if any questions/ concerns, checking in with family, etc In your ideal world – what would the process of transition from pediatric to adult care look like? |

^1^ The caregiver and adult provider interviews addressed similar topics as those presented above. Additional interview guides, consent forms, and other study materials are available from authors upon request.

**Supplementary Table 2: Additional quotes highlighted in the thematic analysis.**

This table contains additional quotes supporting the thematic analysis, demonstrating a broader sampling of participant perspectives than we were able to fit within the limited space of the manuscript.

**Theme 1: Leaving the pediatric comfort zone: autistic patients navigate healthcare without guidance**

| Subtheme | Topic | Quote |
| --- | --- | --- |
| 1a. Agency and healthcare decision-making | Expectations of adult care | [Adult care is] a lot more independent, and we’re one-on-one instead of sort of talking through a parent. (Post-transition patient)  “ [I]f he still needs me to go with him, that’s fine, but at least he knows what to expect and what’s being expected of him. That’s key right there, is that I want him to know what he needs to do and what’s the appropriate behavior and reaction to whatever the situation might be, because I don’t want him to overreact…” (Caregiver of pre-transition patient) |
|  | Disclosure of autism | “Well, I have a good bunch of good friends, and I’m a good student at [name of high school]. I study a lot, and I like to go to the gym to workout… Maybe they can know about my autism and stuff like that” (Pre-transition patient)  “I’d want my doctor to know about that, like, in the high school years, all of my injuries were rugby and stuff, and then also my probably ADHD, being on the ASD spectrum.” (Post-transition patient)  “I always worry that they don't know she has autism… when they pull up her chart…And some people tell me, "No, they don't," because that's personal. But I'm, like, "I need for them to understand why she says certain things, you know.” (Caregiver of post-transition patient) |
|  | Navigating health conservatorship and supported decision making | “One family … they almost kind of cornered him… I said, "Well, I'd like to meet with everybody together. I'd like to speak specifically with him and just see if he understands what's going on…you know, I just didn't really know if he really fully understood the ramifications” (Adult Provider, speaking about a sterilization procedure).  “I think with anyone turning 18 there’s that privacy…I’ll just bring it up, that “There’s legal change that happen after 18.” And fortunately, I think the families that have gone through that process have been savvy enough to kind of navigate that on their own” (Adult Provider).  “If there was some way that [name of medical facility] could set it up for a person with a conservatorship, that both of them got emails… It’s, like, I had to set it up so it’s all coming for me, right? So I set myself up as the person – there doesn’t seem to be any alternative” (Caregiver of post transition patient). |
|  | Provider communication styles facilitating accessible healthcare | He [provider] kind of spoke plainly to me… He talked to me, primarily, instead of [to] my parents. (Post-transition patient)  “it takes me, like, a while just to process information, so it may take me longer for me to answer. It’s not, like, that I’m ignoring the person. It’s, like, it takes me a while to…understand… what they’re telling me before I can give an answer” (Post-transition patient).  “So, I think it's more the listening and just asking her a lot of questions to really figure out, "Okay. Where does it really hurt?," and, you know, "Point to where it really hurts," and stuff like that. It's just patience with her” (Caregiver of post-transition patient).  “I would probably speak a little bit more slowly and more clearly and more simply – make sure everybody in that room understands. Also making sure that I give time for the patient to communicate…Just have to give them the time to allow them to communicate” (Adult Provider). |
|  | Flexible and accessible terminology and questions | “…the patient can use their own terminology and stuff… sometimes it feels like doctors have a very narrow definition, you know, have a very specific terminology for different things that aren’t necessarily, you know, like connecting with me… just like, “Is what you’re experiencing X or Y or Z?” And…say like, X, Y, and Z don’t feel like they’re describing what I mean, you know?” (Post-transition patient)  “If I’m experiencing pain or something, it’s harder for me to narrow it down to a particular place. Or, like questions about, like, what type of pain it is. That’s always been really difficult for me to answer, historically. You know, I mean, is it a throbbing pain? Is it a dull pain? I just don’t know” (Post-transition patient). |
| 1b. Transition readiness | Patient engagement and interest in transition | “Because they were an adult doctor, and like I’m 18. I thought I was ready to talk to, like, an adult doctor. (Post-transition patient)  “I: did you get a letter in the mail or an e–mail or anything that told you, "… It's going to be time to change to someone new." Patient: No, actually, I didn't get a letter in the mail or an e–mail. My mom might have…on that point, I wasn't very good at picking out that kind of stuff. So, mainly, I just let my mom handle that kind of stuff…” (Post-transition patient). |
|  | Assessing self-efficacy and independence in transition | “We’re going to have to navigate through that area [healthcare], because I know that the second that he turns 18 he’s not going to be able to do it all by himself. But, you know, as an 18-year-old, developmentally-wise, he’ll probably be 14 or 13 or whatever.” (Caregiver of pre-transition patient)  “She’s supposed to be calling and advocating and all that for herself, and getting on the computer and stuff. That’s not happening all the time. I’m on her bottom, you know, saying, “Do it, do it, do it, do it!” She’s still not quite prepared to be on her own, and we are trying to get her that way.” (Caregiver of post-transition patient).  “I think that’s a gut thing; depending on their response to conversations or suggestions that we may have made, and then seeing both the efficacy of follow-up and/or not… in general…unless the family’s asking for aid or other support in that realm, I don’t often go there…” (Adult Provider). |
| 1c. Changing role of parents | Parent shifting to supporter | “They both listened … I think that was really big for [name of child] …they kept saying, "…if you want to talk alone" …it kind of tried to make him become more independent – which I thought was a great thing. They were trying to have him kind of come out of his shell. Because he relies on us a lot” (Caregiver of post-transition patient).  “I would definitely always involve the patient, but I would also – you know – I also try to involve everyone in the room.” (Adult Provider). |
|  | Parent as expert and advocate | “Early on…as a parent, you quickly become the expert on that [autism]. So, there was very little guidance, and in a lot of ways, there was more guidance from me going to the caregiver or to the medical staff about how to successfully be able to interact with, manage, whatever.” (Caregiver of post-transition patient) |
|  | Parent as primary navigator of health system and services | “Asperger’s variety autism…is a communication disorder…so, when you’re trying to navigate a system like [name of medical facility] and you’re frightened and nervous and you’re cranked up with anxiety and you don’t know what to expect and you can’t find any information, my son won’t go looking. He won’t know about a business office or member services center…” (Caregiver of pre-transition patient). |
|  | Parent as manager and communicator inside and outside the clinical visit | “A lot of times, he wants me to still come in for that [adult care visit]... I think he just feels more comfortable having somebody that he knows. He doesn't like the unknown” (Caregiver of post-transition patient).  “Yeah, she[mother] used to [attend appointments], but she doesn’t anymore … It was nice to have someone who was able to, like, convey what I was saying a little bit easier, I guess” (Post-transition patient).  “He gets it in the room, but when we go home, it doesn’t generalize to the rest of the world or his life. So, I just want to say, you know, it’s pretty much up to the parents to carry it through” (Caregiver of pre-transition patient).  “Sometimes, for some of my patients, I'm not actually really communicating much with the patient at all, but more with the family – more with the mom or the dad, because of the condition with the patient…Sometimes, it's a joint situation” (Adult Provider).  “In my experience, most of the kids who are coming through from pediatrics have really been set up quite nicely in the medical/social skill or area. I haven’t noticed a patient with a lack of social support or some social need…or didn’t have somebody helping them to work on” (Adult Provider). |
| 1d. Transition beyond healthcare | Desire for information and community resources | “I don’t know if doctors have connections to certain career programs… connections for disabled people…so the program can give these people jobs” (Post-transition patient).  “The adult doctor really hasn’t suggested, “[Name of participant] should go to this group; [name of participant] should go to this group.” I don’t even know if there is a group, you know… they need to tell the doctors to suggest groups for these autistic people. If they have high blood pressure… they need a life skills class and they need an exercise class…stuff like that” (Caregiver of post-transition patient).  “in an ideal world, there would be a provider that really understands, sort of, how to navigate everything from…disability support programs at the community colleges in the area to supported employment programs … I think that’s more important than any kind of immediate concrete psychiatric help is honestly helping families to link to services” (Pediatric Provider). |

**Theme 2: Health consequences of a passive healthcare transition**

| Theme | Subtopic | Quote |
| --- | --- | --- |
| 2a: Little support to proactively engage in transition | Resource cliff, drop in pediatric services | “As they become older… it seems like a lot of resources fall to the wayside… you can still go to a psychologist, and you can go to a group if you have anxiety…but socially, it's very hard, because they're still…roughly two years behind socially… how do you date? You know, how do you balance things?... it just all kind of just goes away” (Caregiver of post-transition patient).  “The transition of leaving high school, of suddenly, your whole life has changed, overnight. There’s no structure. There’s no social life and all those people that you knew – for him, hundreds of people, and all those adults he related to every day that gave him support all day long, they’re not in the picture.” (Caregiver of pre-transition patient)  “There doesn’t seem to be any treatment for adults with ADHD, and so, I don’t even know what there is for people with autism. If they won’t even help people with ADHD, what hope do autistic people have? (Caregiver of pre-transition patient) |
|  | No systemic transition supports for physicians | “I'm actually not even certain that we have protocols installed for patients that transfer for pediatrics to adult medicine that have an autism spectrum diagnosis.” (Adult Provider).  “the pediatricians, some of them, said, “You know, we don’t even know when some of our patients transition”  They graduate and they turn 18, and it automatically gets done, and the pediatrician doesn’t know… Could we identify patients who have like autism and send the pediatrician a letter saying, “Hey. You know, this patient is going to be graduating in the year.” You know, “Think about reaching out” (Adult Provider).  “Every answer I give around the stuff is in the context where all mental health clinicians in all [name of medical provider facilities], as far as I’m concerned, are completely overburdened with having way too many patients– so you’re just trying to keep your head above water, focusing on the crises. So, it can be really hard to find the time to initiate a conversation [re: transition)”. (Pediatric Provider) |
|  | System does not engage patient and parent | “…the medical system drops them and it’s, like, “There’s still a problem, people!” You know? … don’t drop them. They can succeed” (Caregiver of post-transition patient).”  “We really haven’t accessed any sort of pediatric primary care since then. I mean, the occasional illness, infection… we were seeing [Specialist] so frequently, you know, we didn’t have much occasion to utilize a primary pediatric care… I felt like we got all the care and attention we needed from that team, from the pediatric endocrinology team” (Caregiver of post-transition patient). |
|  | Logistical/appointment management support (System level constraints) | “…I understand there's needs for patient-driven access, first-call booking, this and that. There are a lot of numerics. And that's unfortunately – the first appointment of a special needs patient…there's no numeric for that… And hence, there's not really necessarily an incentive on the part of the physician to really necessarily spend the time that they might need to really get to know the patient. It's not really the physician's fault” (Adult Provider). |
|  | Technological supports and alert fatigue (System level constraints) | “best practice alert… that pops at the age 16…if someone has a special need, it – maybe it should pop up sooner… And so, during the course of whatever the reason that they come in for, the pediatrician should also bring up, "By the way, Johnny is 16 now, and quite soon, he will be legally an adult. And we have to think about the future of his health care.” (Adult Provider).  “the pediatrician is supposed to review the list and say, ‘Aha!…There's a patient with autism spectrum disorder – has these needs’… So, the problem…there's a lot of things that come into people's desks and in-baskets… the pediatrician may have it and decide, "I'm going to look at this later,"…Or some of them, unfortunately, just glance at it and then toss into recycling” (Adult Provider). |
|  | Lack of training and interest (System level constraints) | “… because there’s such a gross lack of interest or ability to work with that population in the adult psychiatry in [location of medical facility], it really hasn’t been much transition happening” (Pediatric Provider).  “…the lack of interest could stem from a lack of knowledge… So, I wouldn’t want to treat certain conditions in child psychiatry because I don’t have any experience” (Pediatric Provider). |
| 2b. Episodic healthcare | Lack of regularity adversely impacts transition | “identifying the patients who might need a transition, because they slip the cracks… you don’t know, their last visit might be when they’re like 16-1/2 and they’re here for a cold, and they don’t come in to see you…especially with the autistic patients, some of them do not come in that often” (Pediatric Provider). |
| 2c: Sexual Health: Who should have “the talk” with the patient? | Limited discussion of sexual health with Autistic adolescents | “We were just discussing … the sexual issues with the mom. She is – very limited communication – it's kind of like yes and no. That's basically it. So, it's hard for me to usually gauge how much she actually understands or doesn't” (Adult Provider).  “It’s a very difficult conversation to have with these kids, because there’s that idea of – you know, there’s sex good and there’s sex bad. She [gynecologist] was very good at explaining that to [name of patient] and talking to her about getting pregnant and, you know – it was a good conversation. I was a nervous wreck. That was one of my – oh, my God! I had anxiety on that one, but it was a very good experience. I have to say it was a very positive experience. She was very good with [name of patient]. She took a lot of extra time, I have to say. (Caregiver of post-transition patient) |
|  | Uncomfortable topic | “the whole thing about their sexual activity is, like, I’m not really into that, so that probably made me a little bit uncomfortable” (Post-transition patient). |

**Theme 3: Strategies for inclusion and continuous engagement**

| Theme | Subtopic | | Quote |  |
| --- | --- | --- | --- | --- |
| 3a. Gradualism | Fluid timing for change from pediatric to adult care providers | | “Pediatricians…should be – continue to be the primary care doctor for the patient up to the age of 21 …when you have these very high-need patients, that then they may need transition or change, or even when they turn 18, they may not be until 21” (Adult Provider). |  |
|  | Begin transition process early | | “start it [Transition planning] earlier than it has to happen, because the abrupt ending of pediatric care is emotionally kind of a hard thing to do. It’s like, “Here’s somebody that you’ve seen for your whole life, and now you’re not going to see them anymore.” So, maybe, let there be almost sort of a ceremonial end to it and introduction.” (Caregiver of post-transition patient)  “…[Be]cause these kids, you know, some of them have so much difficulty with transition. Fortunately, [patient name] is not one of those kids, for the most part. As long as he has enough advanced warning; he will sail through any transition, as long as he knows it’s coming. (Caregiver of a post-transition patient)  “I think they [healthcare facility] handled it [the transition] really well. I think there was enough warning, this is going to happen, this is going to happen. And, so that … – he [the patient] realized there was going to be a change, … – and then, the [post]card came in [announcement of transition to family medicine]. The whole thing, I think it was good, because it gave him time to accept the change.” (Caregiver of a post-transition patient) |  |
| 3b. Warm handoff | Proactive communication between providers | | “at least half of it, should come from the pediatrician… If they’re on the autistic spectrum, transition is hard. So I think it would be great for them to help them find a new doctor. And that way, they can also give some information about the new doctor and reassure …that [name of adult physician] was great, that he knew him, and that he was really kind”(Caregiver of post-transition patient).  “Hopefully, when they do, our hope is that there has been some type of clinical summary provided by the pediatrician, which could be in the problem list, or sometimes they send over a staff message” (Adult Provider).  “I wish there was a better process for chronic kids that allowed for the time, you know, so whether or not it’s a roundtable, but for, a handoff for the pediatrician, the adult [U/I 20:38] that the patient, you know, could all be involved and just review the case… for conversations and things like that to happen” (Pediatric Provider).  “Ideally, if there could be that transitional call … from the pediatrician… so that they can say, “This is who I would like you to see” or “Who you’re going to see” and “I’m going to reach out to them and speak specifically to them.” I think that would be great.” (Adult Provider). |  |
|  | Personal touch | | “She wrote him kind of a goodbye letter, you know, talking about it. And it was really nice, and it meant a lot to [name of child]…congratulating him, you know, so it wasn’t a “I’m done with you. See you.” It was congratulating him for kind of graduating from the child pediatric – the child psychiatry to adult psychiatry, and how much she enjoyed working with him, and good luck with your next one…it was all like that, really positive (Caregiver of post-transition patient).  “He [Pediatric provider] said…‘You’re an adult now, [name of child], and, you know, you’re 20, and you’re going to start seeing a new doctor. And I’m still going to be working here, but it’s time for you to start seeing a new doctor, and he’s really nice. And, you know, I’ve talked to your mom about him, and I think he would work really well with you.’ And he was very reassuring” (Caregiver of post-transition patient). |  |
| 3c. Clinical tools and accommodations for patients | Videos and priming | “Having them look at a video would be very helpful…because it kind of takes that anxiety out [by letting autistic patients] know what to expect. There’s already anxiety going to the doctor.” (Caregiver of pre-transition patient).  “I think an email would be really good. It’s like, “Here’s the appointment preparation checklist.” … If they’re going to be taking labs, is he going to be expected to pee in a cup?” in general, the order that things happen when they come in…” (Caregiver of post-transition patient)  “[The healthcare system should] put out a pre-appointment list [for caregivers]: These are the things you might talk with your adult child before you get here: 1. We’re going to weigh you, 2. We’re going to check your blood pressure, 3. And then, you’re going to go into the exam room , 4. You may have to wait a little while, 5. You may have to get undressed.” (Caregiver of a post-transition patient) | | |
|  | Lists of providers | “We don't have the list, but that would be great! [laughs] If we had a list of providers that have a special interest in that and are more knowledgeable on patients with autism, that way they'd probably be better taken care of” (Adult Provider).  “…if there was something that tells these [providers] some sort of idea about how to act around special needs people– can you tell if somebody has experience with people with autism?...If there was more of a list available” (Caregiver of post-transition patient).  “Most of my research is among the parent community. I ask my friends “Is there anybody that you would recommend as an adult medicine provider, you know, that … has either patients with or (laughs) interest in dealing with adults with disabilities?” (Caregiver of a post-transition patient) | | |
|  | Lists of resources/post-visit engagement tools | “a follow-up email or something, and making sure – following up with her to make sure that she is following the protocol that the doctor suggested, would also probably be a good idea… It would need to be a bolder text and simple. Definitely bolder, more white space. Yeah, those would not be sufficient, I don’t think… if the doctors can even send video, but something like that would be a good” (Caregiver of post-transition patient).  “…in terms of a bundle of tools, I don’t think doctors are really that good about knowing – […] about SSI and some of the confidentiality; if people want that information to be, like, a package to be able to give their patients or a better understanding of that, but I don’t think that, from a pediatric standpoint or a physician’s standpoint, that we feel like we know the information that we’re supposed to be providing to the patient and their families (Pediatric Provider).  “They have an appointment…and a new patient alert pops up with a little yellow banner… it does have some printable information and, if they’re 18 to 25, a couple of informational printables that you can get to the family…ways to use the system in an easier way – resources for the family” (Adult provider). | | |
|  | Alert for clinic staff | “If there was some way to mark off that these people are going to need extra help communicating. Something on that chart for even the person at the front desk, you know, this is a communication for her, for my daughter” (Caregiver of post-transition patient). | | |
|  | Technology not enough for long term independent management | “And he has gotten test results on [provider website].org, he’s gotten emails from the doctors. I’ve tried to show him how to go in and check it. And he’s very computer literate; he has the ability to do that, but he doesn’t necessarily have the interest to do it…He doesn’t care! He’s not going to go in and look at them… I want to say it doesn’t have any meaning to him.” (Caregiver of post transition patient) | | |
|  | Transition appointments | “Patients are going to need more than just 20 minutes…especially for a first visit. And that first interaction is just, "Hi. Oh, I see, I read your chart. And let me just examine you. And okay, goodbye…’ Then, it's not going to be a very good interaction… there's been some discussion about, "Well, for these special needs patients, that first visit should be maybe a double appointment – a 40-minute visit” (Adult Provider).  “So, in terms of adjusting the time, I really – I can't adjust the time, because the appointments are set. And that's why I tend to sometimes just run behind. Because, again, there's no adjusting appointment times” (Adult provider)  “[I]t’s not because they’re not capable of speaking. You know, it might just be that, you know, they’re shy in doing so. They need more time to get warmed up; they need more time to get comfortable. And sometimes they might get overwhelmed by too much information. (Caregiver of a pre-transition patient)  “I think everybody wishes for more time. The time to be able to do it [the transition] well. (Pediatric provider) | | |
| Tailoring/personalizing care for autistic youth | Transition class/info video for autistic youth | | “I think that all the kids that are transitioning or maybe prior to that, they all need to be put in a group or some kind of a classroom-type thing and have some kind of an intense thing, training, about life skills, about practical things … taking charge of your medical problems and how do you do that through [name of medical facility] … You know, how do you, you know daily living kind of skills” (Caregiver of post-transition patient). |  |
|  | Medical home/care coordinator | | “I really think that [name of service provider] needs to have a team in place that calls, and checks on these people… when they’re supposed to come in and have a well check, they’re going to need to have some kind of call or follow-up that says, “Hey, this is nurse so-and-so. Remember me... It’s time for you to come in” (Caregiver of pre-transition patient).  “I think there should be like a transition coordinator of sorts who kind of reaches out to the pediatrician, to me, and then to the adult provider, and has like a transition plan or gathers the relevant information because in the context of us, again, not having enough time to really proactively do a lot of the stuff we’re talking about in this ideal transition plan” (Pediatric Provider). |  |
